# Unraveling the neural basis of spatial orientation in arthropods

**DOI:** 10.1007/s00359-023-01635-9

**Published:** 2023-05-17

**Authors:** Uwe Homberg, Keram Pfeiffer

**Affiliations:** 1 Department of Biology, Animal Physiology, Philipps University Marburg, 35032 Marburg, Germany; 2 Center for Mind Brain and Behavior (CMBB), Philipps-University Marburg and Justus Liebig University Giessen, 35032 Marburg, Germany; 3 Behavioral Physiology and Sociobiology (Zoology II), Biocenter, University of Würzburg, 97074 Würzburg, Germany

## Abstract

The neural basis underlying spatial orientation in arthropods, in particular insects, has received considerable interest in recent years. This special issue of the *Journal of Comparative Physiology A* seeks to take account of these developments by presenting a collection of eight review articles and eight original research articles highlighting hotspots of research on spatial orientation in arthropods ranging from flies to spiders and the underlying neural circuits. The contributions impressively illustrate the wide range of tools available to arthropods extending from specific sensory channels to highly sophisticated neural computations for mastering complex navigational challenges.

## Introduction

Anyone who has observed honeybees flying in and out of their hive collecting nectar and pollen on a sunny spring day cannot but wonder what guides these insects to a particular patch of flowers and, when loaded with food, back to the hive. Mobile organisms such as these bees and other animals usually do not move arbitrarily within their environment, but in ways that are likely to lead them to desired or favorable places or, alternatively, away from unfavorable or dangerous spots. Evolutionary forces have shaped and refined the neuronal machinery underlying these behaviors to enable and control them. Arthropods and vertebrates arguably encompass organisms with a high degree of mobility. Both taxa comprise species that are able to use sophisticated mechanisms for spatial orientation in their home range, the area in which the animal lives and moves on a regular basis, but also to find home after displacement to unknown sites. This latter capability requires positional information (“map” sense) as well as directional information (“compass” sense) and is often termed “true navigation” which has been proposed for amphibians, reptiles, birds, but also spiny lobsters (Phillips et al. 2006). It allows animals to perform long-range seasonal migrations between areas not visited before. Seasonal migrations are common in many flying insect species (Hu et al. 2016; Florio et al. 2020) with monarch butterflies migrating from the United States and Canada to overwintering sites in central Mexico and dragonflies crossing the Indian ocean being the current record holders (Reppert and de Rohde 2018; Hedlund et al. 2021). Within home ranges, various forms of spatial learning and memory play major roles in navigational performance both in vertebrates and in arthropods (Collett et al. 2013; Murray et al. 2016; Pfeffer and Wolf 2020). These examples suggest that certain arthropods may compete favorably with the navigational capabilities of vertebrates, and what is particularly astonishing, they achieve this with a brain size of only a tiny fraction of that of a vertebrate. Research on navigational behavior and its underlying mechanisms in arthropods has, therefore, a particular fascination for many neuroscientists, not least because it may facilitate insights into minimum network requirements underlying the observed navigational performance and also provide attractive solutions for robotics applications.

Analyzing the neural control of a particular spatial behavior has many facets. It requires detailed understanding of the behavior itself, its ecological adaptations and limitations, its cognitive requirements, and its benefit for the organism. In addition, it requires analyzing the sensory systems involved, ranging from characterizing sensory receptor cells, interaction of different sensory systems, to central processing pathways and mechanisms. This may, finally, allow for the identification and characterization of brain areas that compute and control navigation and, based on information on neuronal circuits, facilitate computational models reflecting the observed neuroarchitectures and behavioral performances. Considerable progress in all of these areas has been made over the last two decades, most prominently perhaps the detailed analysis of head direction coding in the central complex of *Drosophila melanogaster* (Seelig and Jayaraman 2015; Hulse and Jayaraman 2020) and, more recently, the ability of the fan–shaped body to perform computations in allocentric space (Lu et al. 2022; Lyu et al. 2022). The collection of articles in this special issue reviews and highlights particular aspects related to all of these fields of research on navigation control in arthropods.

## Contributions to this special issue

This special issue is composed of eight original research articles and eight reviews covering a broad range of specific aspects of spatial orientation in insects and arachnids. The contributions are loosely assigned to different topics but most of them actually cover additional areas or have implications for other aspects of orientation. In the following, we have tried to highlight the main messages and conclusions conveyed by the authors of the different contributions and put these into the broader context of related findings.

### Navigation by olfactory cues

Olfactory cues are especially important at night and are, therefore, widely but not exclusively used by nocturnal species. In their review, Theresa Steele and coworkers point out that odor source localization from an odor plume or a chemical trail is not a trivial behavior but requires sophisticated temporal integration and spatial search strategies such as casting behavior (Steele et al. 2023). For determination of flow direction, integration with wind or optic flow sensors becomes important. Neural circuits involved in olfactory navigation have been worked out to some extent and point toward the mushroom bodies and, secondarily, the central complex/lateral complex as major sites involved. Of particular significance are recent findings in *Drosophila*, reviewed by Steele et al. (2023) of antennal input into the head-direction system of the central complex, suited to represent the neural basis of search and casting behavior.

### Vision and view-based navigation

Vision is, without debate, of primary importance for a multitude of orientation behaviors. The sensory basis for visual orientation is provided by the eyes. The physical properties of the dioptric apparatus and the physiological properties of the photoreceptors define the limits of the visual information that can be perceived. Yuri Ogawa and colleagues investigated the anatomical and physiological properties of the compound eyes of the green weaver ant (Ogawa et al. 2023). The ants are diurnal and use vision for navigation and prey capture. Using pattern electroretinography, the authors show that, for a walking animal, green weaver ants have a relatively good temporal resolving power suggesting that their visual system is well adapted for bright light conditions. Their spatial resolving power allows them to resolve fine details in a visual scene. The small number of ommatidia together with their small size results in lower contrast sensitivity when compared to other predatory ant species. The results, taken together, fit well to the visual ecology of this slow-moving diurnal ant species.

Jochen Zeil reviews the involvement of the visual system in forming navigational memories of panoramic views which are important for following routes and essential to pinpoint particular locations (feeding sites, burrows) in space (Zeil 2023). This is especially but not exclusively important for central place foragers such as bees, ants and wasps. Memories of landmark panoramas are recalled by a process called view matching, whereby heading direction is achieved by evaluating differences between stored panoramic views with currently experienced views. It is currently unknown, how and where such views are represented in the brain. Based on place learning experiments in *Drosophila* (Ofstad et al. 2011) and its coarse visual filter properties, Zeil suggests that the central complex may be a promising site for matching stored and current views.

Radar tracking of honeybee flight paths associated with foraging performed by Randolf Menzel and coworkers provides deeper insight into the memory structure formed in the bee brain. Menzel (2023) argues that landscape memories are principally different in flying insects like bees from walking species like ants. From their aerial views, bees may be able to form memories from landscape structures through sequences of overlapping views while walking insects like ants basically have to rely on 2D panoramas. Various forms of displacement experiments show that honeybees are able to perform sophisticated short cuts to their nest, suggesting that they have knowledge about their position within their home range that goes beyond pure vector calculation as demonstrated, e.g. for navigating ants. These experiments also argue that experienced dance followers in the hive do not exclusively rely on vector information obtained from the dancing bee but, in addition, use their own landscape memory to find the feeder. How combinations of vector memories derived from a sky compass and landscape memories are stored is currently unknown but, as suggested by Menzel (2023), may involve large parts of the brain, including the optic lobes but, in particular, the combinatorial power of the large mushroom bodies. Visual cues that guide spatial orientation in bees include sky-compass cues and the distal visual panorama, but also local cues like landmarks or the color of a flower.

Norihiro Kobayashi and coworkers investigated the role of octopamine in visual orientation learning by combining a closed-loop flight simulator with appetitive color-conditioning of the proboscis extension response (Kobayashi et al. 2023). They show that honeybees, which have been conditioned to the color of a rectangle, will preferentially orient toward the learned color in a newly developed closed-loop flight apparatus, and that this memory persists at least 24 h. Application of epinastine, which blocks octopamine receptors, abolished the orientation response, indicating an involvement of octopamine in this form of learning.

### The role of optic flow information

The importance of optic flow information for proper spatial orientation, especially in fast flying insects, is thoroughly reviewed by Martin Egelhaaf (Egelhaaf 2023). The author illustrates the crucial role of optic flow signaling for collision avoidance, during landing, in landscape learning, when negotiating gaps, and in distance estimation during path integration. The complexity of visual flow information is often reduced by the animals by a separation of translational and rotational flow fields through alternating saccadic movements and gaze periods. Flight speed is usually adjusted to keep the flow field at a constant level and, accordingly, is reduced when the animal flies through a narrow gap. Egelhaaf discusses the requirements needed, if estimates of distances are extracted from optic flow information as has been shown for bees. The processing of optic flow information has been studied in depth within the optic lobes, especially in flies. Signals are fed directly to descending pathways, allowing to maintain flight balance or initiate escape responses. In addition, several other brain areas receive optic flow input, among them the central complex, likely combining distance and directional information for path integration as suggested by Stone et al. (2017) for sweat bees.

In search for a neural pathway providing optic–flow derived motion information to the central complex, Anna Honkanen and coworkers have studied interneurons in the bee’s central brain sensitive to optic flow stimulation (Honkanen et al. 2023). The authors provide a plethora of single-cell recording and labeling data of neurons responsive to translationally and rotationally moving gratings in the sweat bee brain. In addition, receptive fields were measured with a single moving stripe. Some of these neurons have been studied previously in the honeybee, other are completely novel. The data show that numerous brain areas receive optic flow information. A particular type of lobula projection neurons with central projections partly overlapping with the dendrites of noduli input neurons most closely matches the signaling properties of nodular inputs to the central complex (Stone et al. 2017). The paper by Honkanen et al. (2023) elegantly illustrates how neural pathways in the insect brain serving particular navigational purposes can be untangled and characterized.

### Learning navigational cues

When first leaving the nest, bees and ants show characteristic learning flights, resp. walks. Two contributions, one from Olivier Bertrand and Annkathrin Sonntag on bees and the second by Wolfgang Rössler and coworkers on desert ants, illuminate research to uncover the neural mechanisms of and changes in the brain during first excursions of these insects outside the hive/nest (Bertrand and Sonntag 2023; Rössler et al. 2023). Bertrand and Sonntag summarize evidence that multisensory cues contribute to familiarize a bee with the surroundings of the hive, including visual landscape and sky-compass information, and non-visual cues such as olfactory or magnetosensory information. During learning flights bees continuously increase their distance to the hive and perform frequent home flights, likely to calibrate their path integrator before becoming experienced foragers. Observations on bees and bumblebees are compared and illustrate slightly different strategies of spatial memory formation.

Learning walks by desert ants show a similar structure (Rössler et al. 2023). In these insects the earth’s magnetic field serves a role as an earthbound compass, against which a sky compass appears to be calibrated. In addition to an outline of behavioral observations during learning walks the authors also report on changes in the brain when ants leave the nest for the first time. The results show that structural changes in synaptic circuits along two visual pathways are different following passive sensory exposure and after the active performance of learning walks under natural sky light. Exposure to sky-compass cues is apparently involved in an increase in the number of synaptic complexes in the collar of the mushroom body calyces and in the volume of the central complex. These data provide important hints at candidate storage sites for panoramic view-based memories in these insects.

A particular behavior of desert ants, often seen at the start of a walk, are fixations interrupted by saccadic body turns. These movements likely aid in panoramic view matching and deciding which path to follow. Sudhakar Deeti and coworkers have analyzed these saccades in detail to derive hypotheses about how they may be generated in the brain (Deeti et al. 2023). They show that this behavior has a particular temporal structure suggesting an underlying random-rate process. The authors discuss the involvement of mutual interactions of the lateral accessory lobes in oscillatory movements, but whether this circuit or input from the mushroom bodies is also involved in the observed body turns of ants requires monitoring neural activity in the navigational circuits in the ant’s brain.

### The central complex network

A detailed review on brain areas and neural mechanisms that likely underlie sky-compass navigation in a seasonal migratory insect, the desert locust, is given by Uwe Homberg and coworkers (Homberg et al. 2023). Over the past 30 years deep insights into visual mechanisms and central processing streams underlying sky compass coding in this insect have been achieved and have inspired similar research in other insect species such as dung beetles, the fruit fly, the monarch butterfly, and bees. Most importantly, the central complex was identified as the brain area that encodes, in a topographic manner, head direction through a combination of sky polarization and direct sunlight input (Heinze and Homberg 2007; Zittrell et al. 2020).

Insights into directional coding by central–complex neurons in monarch butterflies are provided by a study of Jerome Beetz and Basil el Jundi (Beetz and el Jundi 2023). The authors have analyzed whether and how spatial tuning of central–complex neurons is affected by different stimulus dynamics, as would occur during turning saccades vs. more slower changes in flight direction. The authors show that compass precision increases with increasing angular velocities of a simulated sun that rotates around the head of the animal and is, likewise, affected when comparing an erratically against a continuously moving sun. The data suggest that differences in the precision of directional tuning may reflect differences in the temporal adjustments of moment-to-moment steering decisions of the insect.

As emphasized in several contributions to this special issue, sky-compass signals are important but by no means the only sensory cues affecting navigational computations in the central complex. This is highlighted by the contribution of Pratyush Kandimalla and coworkers, who made use of the connectome data of the *Drosophila* hemibrain to map in a comprehensive manner all input channels to the central complex in the fly *Drosophila* (Kandimalla et al. 2023). The authors identify and characterize all clonal lineages in the *Drosophila* brain that contribute to large-field tangential inputs to the central complex in addition to those that constitute the columnar neurons. Among these are “major” lineages that contribute a large fraction of their neurons to the central complex, while others contribute only a few neurons. Cell types arising from these lineages and the fiber tracts they use to enter the central complex were identified and likely homologous neurons and cell types in other insect species are pointed out. The data will be highly valuable to deduce general functional properties of central–complex neurons across insects from functional data in the fly.

Essential components of the head-direction circuit in the central complex of *Drosophila* are global inhibitory elements, but their neural substrate has remained controversial. In a computational model, Ning Chang and coworkers analyzed and compared the possible involvement of two tangential cell types, GABAergic ring neurons of the ellipsoid body vs. Δ7 neurons of the protocerebral bridge (Chang et al. 2023). Both models yielded similar general performance but one or the other appears to be superior when testing for robustness, accuracy, speed, and dynamical characteristics of the head-direction system. The authors conclude that both systems of global inhibition likely coexist in the head-direction system of the fly. This study exemplifies the significance of modeling approaches when addressing the contribution of particular elements to the performance of the entire network.

A direct experimental approach to address the role of the central complex in view-based navigation was undertaken by Cornelia Buehlmann and coworkers on wood ants (Buehlmann et al. 2023). Ants were trained in a white arena to a feeder located to the side of a black rectangle for which they show an innate preference. Mechanical lesions to lateral parts of the central body did not affect the preference in naïve ants for the black rectangle but severely affected orientation to the learnt feeder. As similar effects had been found in an earlier study employing lesions of the mushroom body (Buehlmann et al. 2020), the authors speculate that the central complex lesioned animals might no longer be able to use mushroom body-derived memory for proper orientation.

### Homing and path integration in arachnids

Owing to considerable difficulties in physiological experimentation and lack of model systems amenable to genetic manipulations, research into neural mechanisms underlying navigational strategies in non-insect arthropods is still highly rudimentary despite evidence for highly sophisticated navigational behavior. The review by Joaquin Ortega-Escobar and colleagues shows that homing and path integration is a common navigation behavior in various arachnids (Ortega-Escobar et al. 2023). Ablation studies showed that proprioceptive information, visual input, including celestial compass information and, in amblypygids, olfactory signals are exploited for navigational purposes but central nervous mechanisms have not yet been explored.

## Final remarks

The collection of papers in this special issue highlights the multitude of approaches and research topics associated with endeavors to uncover the neural mechanisms of navigation behaviors on many different levels and in diverse organisms. Behavioral evidence together with physiological and anatomical studies on a variety of insect species repeatedly point to the mushroom bodies and central complex as key sites for navigation memory and computations, but in many instances hard data supporting these claims are still missing. Progress on these fields in insects has greatly benefited from work in *Drosophila melanogaster* owing to unique genetic manipulations and the well characterized behavioral repertoire of this insect species under laboratory conditions. This progress, however, has often been inspired by work on other species specialized in spatial orientation such as social insects (bees, ants) or migratory species (locusts, monarch butterflies). We appreciate the contributions from all authors and reviewers to this special issue and hope that this collection of papers will inspire efforts to further uncover neural mechanisms underlying view memory, path integration, homing and goal directed locomotion, including those in highly understudied arthropod groups, such as chelicerates, myriapods, and aquatic crustaceans.
