# Correction to: Unraveling the neural basis of spatial orientation in arthropods

**DOI:** 10.1007/s00359-023-01655-5

**Published:** 2023-07-12

**Authors:** Uwe Homberg, Keram Pfeiffer

**Affiliations:** 1 Department of Biology, Animal Physiology, Philipps University Marburg, 35032 Marburg, Germany; 2 Center for Mind Brain and Behavior (CMBB), Philipps-University Marburg and Justus Liebig University Giessen, 35032 Marburg, Germany; 3 Behavioral Physiology and Sociobiology (Zoology II), Biocenter, University of Würzburg, 97074 Würzburg, Germany

**Correction to: Journal of Comparative Physiology A** 10.1007/s00359-023-01635-9

In the originally published version of this article, the following three references contained errors and were corrected as indicated:

Beetz JM, el Jundi B (2023) The influence of stimulus history on directional coding in the monarch butterfly brain. J Comp Physiol A. https://doi.org/10.1007/s00359-023-01633-x

The corrected reference reads as follows:

Beetz MJ, el Jundi B (2023) The influence of stimulus history on directional coding in the monarch butterfly brain. J Comp Physiol A. https://doi.org/10.1007/s00359-023-01633-x

Bertrand OJN, Sonntag A (2023) The potential underlying mechanisms during learning flights. J Comp Physiol A 209:23

The corrected reference reads as follows:

Bertrand OJN, Sonntag A (2023) The potential underlying mechanisms during learning flights. J Comp Physiol A. https://doi.org/10.1007/s00359-023-01637-7

Menzel R (2023) Navigation and dance communication in honeybees: ad hoc processes vs memory retrieval. J Comp Physiol A. https://doi.org/10.1007/s00359-023-01619-9

The corrected reference reads as follows:

Menzel R (2023) Navigation and dance communication in honeybees: a cognitive perspective. J Comp Physiol A. https://doi.org/10.1007/s00359-023-01619-9

The original article has been corrected.

